# Modulation of B Cells and Homing Marker on NK Cells Through Extracorporeal Photopheresis in Patients With Steroid-Refractory/Resistant Graft-Vs.-Host Disease Without Hampering Anti-viral/Anti-leukemic Effects

**DOI:** 10.3389/fimmu.2018.02207

**Published:** 2018-10-08

**Authors:** Lei Wang, Ming Ni, Angela Hückelhoven-Krauss, Leopold Sellner, Jean-Marc Hoffmann, Brigitte Neuber, Thomas Luft, Ute Hegenbart, Stefan Schönland, Christian Kleist, Martin Sill, Bao-an Chen, Patrick Wuchter, Volker Eckstein, William Krüger, Inken Hilgendorf, Ronit Yerushalmi, Arnon Nagler, Carsten Müller-Tidow, Anthony D. Ho, Peter Dreger, Michael Schmitt, Anita Schmitt

**Affiliations:** ^1^Department of Internal Medicine V, University Clinic Heidelberg, Heidelberg, Germany; ^2^Department of Hematology, the Affiliated Hospital of Guizhou Medical University, Guizhou, China; ^3^Department of Nuclear Medicine, University Clinic Heidelberg, Heidelberg, Germany; ^4^Division Biostatistics, German Cancer Research Center, Heidelberg, Germany; ^5^Department of Hematology, Zhongda Hospital, Southeast University, Nanjing, China; ^6^German Red Cross Blood Service, Medical Faculty Mannheim, Institute of Transfusion Medicine and Immunology Mannheim, Mannheim, Germany; ^7^Department of Internal Medicine C, Haematology, Oncology, Stem Cell Transplantation, Palliative Care, University Clinic Greifswald, Greifswald, Germany; ^8^Department of Internal Medicine II, University Clinic Jena, Jena, Germany; ^9^Hematology Division, Chaim Sheba Medical Center, Tel Hashomer, Israel

**Keywords:** GvHD, ECP, immunomodulation, regulatory cells, proinflammatory cytokines, effector cells, anti-viral effect, anti-leukemic effect

## Abstract

Graft-vs.-host disease (GvHD), a severe complication of allogeneic hematopoietic stem cell transplantation, significantly affects the post-transplant morbidity and mortality. Systemic steroids remain the gold standard for the initial management of GvHD. However, up to 60% of patients will not sufficiently respond to steroids. Extracorporeal photopheresis (ECP), a cell-based immunotherapy, has shown good clinical results in such steroid-refractory/resistant GvHD patients. Given its immunomodulatory, but not global immunosuppressive and steroid-sparing capacity, ECP constitutes an attractive option. In the case of GvHD, the balance of immune cells is destroyed: effector cells are not any longer efficiently controlled by regulatory cells. ECP therapy may restore this balance. However, the precise mechanism and the impact of ECP on anti-viral/anti-leukemic function remain unclear. In this study, 839 ECP treatments were performed on patients with acute GvHD (aGvHD) and chronic GvHD (cGvHD). A comprehensive analysis of effector and regulatory cells in patients under ECP therapy included multi-parametric flow cytometry and tetramer staining, Luminex^TM^-based cytokine, interferon-γ enzyme-linked immunospot, and chromium-51 release assays. Gene profiling of myeloid-derived suppressor cells (MDSCs) was performed by microarray analysis. Immunologically, modulations of effector and regulatory cells as well as proinflammatory cytokines were observed under ECP treatment: (1) GvHD-relevant cell subsets like CD62L^+^ NK cells and newly defined CD19^hi^CD20^hi^ B cells were modulated, but (2) quantity and quality of anti-viral/anti-leukemic effector cells were preserved. (3) The development of MDSCs was promoted and switched from an inactivated subset (CD33^−^CD11b^+^) to an activated subset (CD33^+^CD11b^+^). (4) The frequency of Foxp3^+^CD4^+^ regulatory T cells (Tregs) and CD24^+^CD38^hi^ regulatory B cells was considerably increased in aGvHD patients, and Foxp3^+^CD8^+^ Tregs in cGvHD patients. (5) Proinflammatory cytokines like IL-1β, IL-6, IL-8, and TNF-α were significantly reduced. In summary, ECP constitutes an effective immunomodulatory therapy for patients with steroid-refractory/resistant GvHD without impairment of anti-viral/leukemia effects.

## Introduction

Graft-vs.-host disease (GvHD) constitutes a severe complication of allogeneic hematopoietic stem cell transplantation (allo-HSCT). Clinically significant acute GvHD (aGvHD) will occur in 40–80% of patients undergoing allo-HSCT (1). Approximately 35–70% of patients will develop chronic GvHD (cGVHD) (2, 3). Systemic steroids represent the first-line therapy. However, up to 60% of patients will not sufficiently respond to steroids and require additional immunosuppressive treatment (4). Broad immunosuppression increases the risk of disease relapse, infections, and subsequent mortality (5). Moreover, the efficacy of allo-HSCT strongly relies on the graft-vs.-leukemia (GvL) effect which is tightly linked to GvHD. Thus, strategies for GvHD treatment that are efficient, steroid-sparing and not compromising the beneficial anti-leukemia and anti-viral immunity are highly desirable.

Extracorporeal photopheresis (ECP) is a second-line treatment for GvHD, which has been associated with good clinical responses. It employs (i) apheresis with *ex vivo* collection of peripheral mononuclear cells, (ii) photoactivation with exposure of leukocyte-enriched plasma to the photosensitizing agent 8-methoxypsoralen and ultraviolet A light, (iii) reinfusion of such physico-chemically modified ECP-treated cells to the patient. In a pooled analysis (6), overall response rates (ORR) were 69% and 64% for acute and chronic GvHD, respectively.

In the case of GvHD, the balance of effector and regulatory cells is severely impaired with effector cells not being efficiently controlled by regulatory cells. ECP therapy might restore this balance. Apoptotic cells play a major role in ECP therapy and trigger the differentiation of monocytes toward tolerogenic dendritic cells. This may result not only in induction of regulatory T cells (Tregs) but also in dysfunction of effector T cells (7, 8). CD4^+^ Tregs and neutrophilic myeloid-derived suppressor cells (MDSCs) (9–13) have been described as cell subsets of importance for response to ECP therapy. However, the immunomodulation of other immune regulatory cells, effector cells and proinflammatory cytokines influencing the success of the ECP treatment remains to be elucidated. This study was performed to address these unsolved questions.

## Materials and methods

### Patients

Twenty patients with steroid-refractory/resistant aGvHD ≥ II and moderate to severe cGvHD received ECP therapy at the University Hospitals Heidelberg and Greifswald in Germany. The diagnosis of steroid-refractory/resistant GvHD is based on the European recommendations (14, 15). Adequate venous access and leukocytes > 1/nl were required to be eligible for ECP. The study was approved by the Institutional Review Board. All participants signed informed consent.

### ECP procedure

Each ECP treatment was administered over two consecutive days using the Therakos UVAR XTS photopheresis system. For patients with aGvHD, 12 weeks of intensive, semiweekly (twice per week) treatment, were followed by biweekly (every 2 weeks) ECP treatment (16, 17). Patients with cGvHD received either an 8-week intensive treatment followed by a biweekly treatment or a biweekly treatment upfront. ECP therapy was stopped when patients either achieved complete response (CR) or maximal partial response (PR) with steroid reduction.

### Sample collection and cell preparation

#### Peripheral blood mononuclear cells (PBMCs) and serum collection

Blood was drawn from consenting patients from the first therapy and every second to fourth ECP cycle before the ECP treatment process. PBMCs were diluted 2:1 with phosphate-buffered saline (PBS), then isolated by density gradient centrifugation (2,000 rpm, 30 min, room temperature, without break) and stored in liquid nitrogen. Serum was isolated (1,500 rpm, 10 min, room temperature) and stored at −80°C.

#### Separation of CD8^+^ T cells and CD8^−^ T cells

After thawing, PBMCs were rested overnight as described earlier (18), followed by CD8 MicroBeads separation according to the manufacture's instruction (Miltenyi Biotec).

#### Enrichment of CD56^+^ NK cells

CD56^+^ NK cells were enriched by negative selection with NK cell isolation kit according to the manufacturer's instructions (Miltenyi Biotec).

#### Fluorescence activated cell sorting of MDSCs

MDSCs subsets were sorted by FACSAria (BD biosciences) using CD11b allophycocyanin (APC) (clone: ICRF44, BioLegend), CD14 APC-eFluor 780 (clone: 61D3, eBioscience), CD33 fluorescein isothiocyanate (FITC) (clone: HIM3-4, BD bioscience), HLA-DR Peridinin chlorophyll (PerCP) (clone: L243, ebioscience) antibodies.

### Flow cytometry

Immunophenotyping and immunomonitoring were performed on rested PBMCs except MDSCs (18). Cells were stained with different combinations of antibodies (Supplementary Table 1). Blocking buffer containing 50% human serum was used to reduce nonspecific binding, and NEAR-IR was used for dead cell exclusion. Each antibody was first titrated to determine its optimal concentration for staining. Appropriate negative controls, fluorescence minus one (FMO) control or un-stimulated control, were used in the study. All acquisitions were performed on an LSRII device (BD Biosciences). To ensure the quality of measurement, CS&T was performed per working day. Furthermore, fluorescence compensation was applied before data acquisition. Data were analyzed using BD FACSDiva software (BD Biosciences). Gate placement was based on the recommendation from the International Multiconsortia Proficiency panel (19, 20).

#### Surface marker staining

Briefly, after 10 min blocking, cells were either stained with antibodies for 15–30 min at 4°C, room temperature (CD33, HLA-DR) or 37°C [C-C chemokine receptor type 7 (CCR7)] in the dark, followed by washing once with FACS buffer (1% bovine serum albumin and 2 mM Ethylenediaminetetraacetic acid in PBS).

#### MHC class I-peptide tetramer staining

After blocking, 1 × 10^6^ cells were incubated with cytomegalovirus (CMV) phosphoprotein65_495−503_ (pp65_495−503_) (CMV-A2)/HLA-A^*^0201 tetramer and CCR7 antibody for 15 min at 37°C, followed by staining with other antibodies for 20 min at 4°C in the dark.

#### Intracellular cytokine staining

Following NEAR-IR (30 min, room temperature, in the dark) and surface marker (20 min, 4°C, in the dark) staining, activated cells which were stimulated with 1 μg/ml staphylococcal enterotoxin B (SEB) (Sigma-Aldrich) in the presence of brefeldin A (Biolegend) for 6 h, were fixed and permeabilized by using Miltenyi Forkhead box proteins3 (Foxp3) fix/perm buffer set, then finally stained for 30 min with FoxP3 and IL-17a antibody.

### Enzyme-linked immunospot (ELISpot) assay

1 x 10^5^ CD8^+^ cells were mixed with auto-CD8^−^ cells at a ratio of 1:1 and plated in triplicate. CMV/Epstein-Barr virus/Influenza virus (CEF) Pool (extended) (JPT Peptide Technologies) was added directly to the experimental well at a concentration of 2 μg/ml/peptide. ELISpot assay was performed according to the manufacturer's instructions, as described previously (21). Image analysis of ELISpot plates was performed with an ImmunoSpot^TM^ Analyzer (Cellular Technology Limited).

### Chromium-51 (^51^Cr) release assay

A 4-h ^51^Cr release assay was performed to test the NK activity, as described previously (22). Briefly, target cells K562 labeled with ^51^Cr were cocultured with effector CD56^+^ NK cells at effector-to-target cell ratios ranging from 50:1 to 6:1. Maximal release and spontaneous release were determined by incubating the target cells with 1% Triton X-100 (Sigma-Aldrich) and medium alone, respectively. NK activity was calculated by the following formula: % specific lysis = [mean count per minute (c.p.m.) (experimental release)–mean c.p.m. (spontaneous release)]/[mean c.p.m. (maximal release)–mean c.p.m. (spontaneous release)] × 100.

### Microarray analysis

Total RNA was extracted with the RNeasy Micro kit (QIAGEN) from sort-purified MDSCs and gene expression determined using Affymetrix GeneChip®; Human Genome U133 Plus 2.0 Arrays. mRNA was amplified and biotinylated with Affymetrix 3′ IVT Pico Reagent Kit before hybridization. Gene Expression Microarrays were scanned using the Affymetrix GeneChip®; Scanner 3000.

### Cytokine analysis

Quantification of the cytokines IL-1β, IL-2, IL-6, IL-8, and tumor necrosis factor-α (TNF-α) in human serum samples was conducted by the LUNARIS^TM^ Human 6-Plex Ophthalmology Kit384 (AYOXXA Biosystems) according to the manufacturers' instructions. Results were analyzed using the LUNARIS^TM^ Analysis Suite Software.

### Statistical analysis

Gene expression was assessed after adjustment by the Benjamini-Hochberg procedure. Differences in cell frequency between before and post ECP therapy were assessed by paired-sample *T* test. The significant difference between healthy donors (HDs) and GvHD patients was assessed by independent *T* test. In all tests, a *p*-value < 0.05 was considered to be statistically significant.

## Results

### Demographics

A total of 20 patients suffering from GvHD were treated by ECP. Nine aGvHD patients were treated with ECP in addition to immunosuppressive therapies including steroids, calcineurin inhibitors and/or mycophenolate mofetil (MMF) as well as pentostatin and/or ruxolitinib (Table 1). Eleven patients with cGvHD received ECP treatment despite triple drug therapy comprising of steroids, cyclosporine A (CsA), mTOR inhibitors, and/or MMF (Table 1). Of these 20 patients, one patient with aGvHD (patients #1) and two patients with cGvHD (patients #15 and #18) had to be withdrawn after only four to five ECP cycles due to pancytopenia, poor clinical condition, or incompliance, which were not associated with toxicity of ECP therapy.

**Table 1:** Patients' characteristics.

**#**	**Gender**	**Age**	**Disease**	**TX**		**GvHD**	**ECP therapy**				**CMV status**		**Infections**
				**Type of TX**	**RIC**		**Post-TX (d)**	**Post-GvHD initiation (d)**	**Duration (m)**	**Cycles**	**D/R**	**Reactivation during ECP**	**Infections during ECP**
1	F	43	CLL	MUD	Y	aGvHD	171	150	1.5	5	–/–	N	N
2	M	59	FL	MUD	Y	aGvHD	150	95	9	25	–/–	N	N
3	F	68	AML	Haplo	N	aGvHD	230	35	< 1	4	–/+	N	N
4	F	62	AML	MMUD	Y	aGvHD	62	41	4.5	17	–/–	N	N
5	M	24	CLL	MUD	Y	aGvHD	195	32	5	13	+/+	N	Mycotic focus in the lung
6	M	68	TPLL	MMUD	N	aGvHD	28	7	2	6	–/–	N	N
7	F	46	CML	MMUD	N	aGvHD	115	22	5.5	16	–/–	N	N
8	F	56	AML	MUD	Y	aGvHD	41	11	1.5	8	–/–	N	TBC
9	F	23	AML	MRD	N	aGvHD	160	117	2.25	7	+/+	N	N
10	M	50	AML	MRD	N	cGvHD	4,285	4,240	27	36	+/+	N	Pneumonia
11	M	68	LL	MRD	Y	cGvHD	1,275	727	60	90	+/–	N	N
12	F	41	DLBCL	MMUD	N	cGvHD	1,290	260	24	42	–/–	N	N
13	M	59	AILT	MRD	Y	cGvHD	1,673	1,529	18	31	+/–	N	N
14	M	66	TPLL	MUD	Y	cGvHD	732	60	6	13	+/+	N	N
15	F	70	AML	MRD	Y	cGvHD	1,214	1,032	< 1	4	+/–	N	N
16	M	54	PTCL	MUD	Y	cGvHD	804	14	>15	27	+/–	N	EBV reactivation
17	M	54	CLL	MRD	Y	cGvHD	600	448	>16	29	+/+	N	Pulmonary aspergillosis
18	M	59	TPLL	Haplo	Y	cGvHD	180	60	< 1	4	–/–	N	Pneumonia
19	M	57	OMF	MUD	N	cGvHD	240	208	12	16	+/+	N	N
20	M	37	CLL	MUD	Y	cGvHD	300	180	10	25	–/–	N	Pulmonary aspergillosis

*#, number; M, male; F, female; CLL, chronic lymphocytic leukemia; FL, follicular lymphoma; AML, acute myeloid leukemia; TPLL, T-cell-prolymphocytic leukemia; CML, chronic myeloid leukemia; LL, lymphoblastic lymphoma; DLBCL, diffuse large B-cell lymphoma; AILT, angioimmunoblastic T-cell lymphoma; PTCL, peripheral T-cell lymphoma; OMF, osteomyelofibrosis; TX, transplantation; RIC, reduced intensity conditioning; MUD, matched unrelated donor; MMUD, mismatched unrelated donor; MRD, matched related donor; Y, yes; N, no; GvHD, graft-vs.-host disease; aGvHD, acute graft-versus-host disease; cGvHD, chronic graft-versus-host disease; ECP, extracorporeal photopheresis; d, day; m, month; CMV, cytomegalovirus; D, donor; R, recipient; -, negative; +, positive, TBC, tuberculosis; EBV reactivation, Ebstein-Barr Virus reactivation*.

All patients showed neither increased susceptibility to infections nor reactivation of CMV nor loss of complete chimerism during ECP therapy (Table 1).

### Development of NK cells without losing NK activity

**Figure 1:**
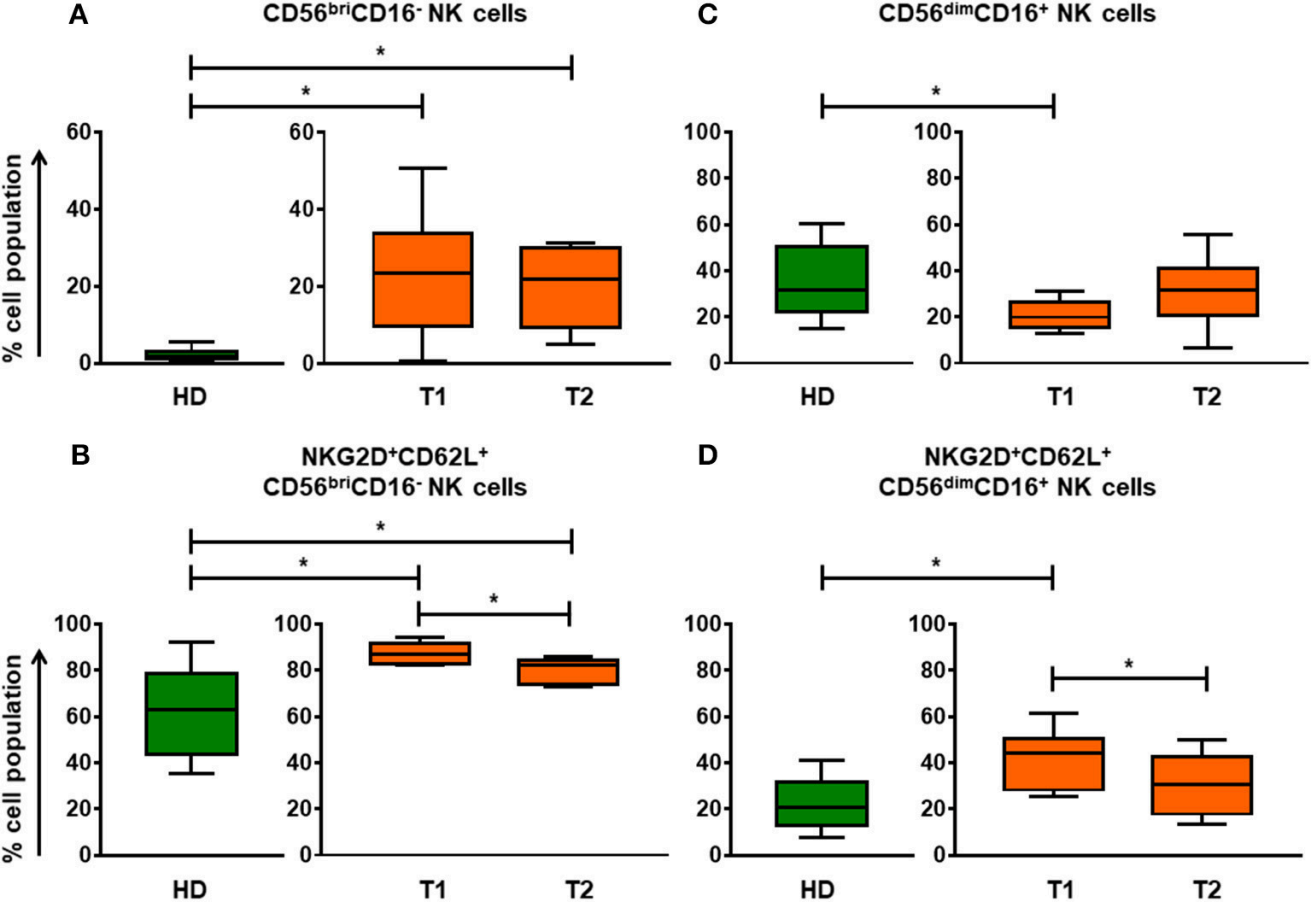
Differentiation and education of NK cell populations by ECP in aGvHD patients. The assessment of CD56^bri^CD16^−^ NK cells **(A)** and CD56^dim^CD16^+^ NK cells **(C)** before and after ECP therapy shows that ECP treatment can promote the development of NK cells from CD56^bri^CD16^−^ NK cells to CD56^dim^CD16^+^ NK cells as well as educate NK cells by decreasing expression of NKG2D and CD62L **(B,D)**. ^*^means *p* < 0.05.

The proportion of CD56^bri^CD16^−^ NK cell subset in aGvHD patients was significantly higher than in HDs (Figure 1A). Undergoing ECP therapy, CD56^bri^CD16^−^ NK cells could slightly decrease (Figure 1A) with significant reduction of the marker expression of NKG2D and CD62L (Figure 1B). In parallel, a normalization (increase) of CD56^dim^CD16^+^ NK cells was observed after ECP therapy (Figure 1C). Moreover, the expression of NKG2D and CD62L on CD56^dim^CD16^+^ NK cells could be significantly reduced by ECP therapy (Figure 1D). To address the question of whether ECP affects the NK activity, we performed a ^51^Cr release assay using isolated CD56^+^ NK cells as effector cells and K562 as target cells. No significant change in NK activity was observed during ECP, as illustrated in Figure 6E.

### Reduction of CD19^hi^CD20^hi^ B cells

**Figure 2:**
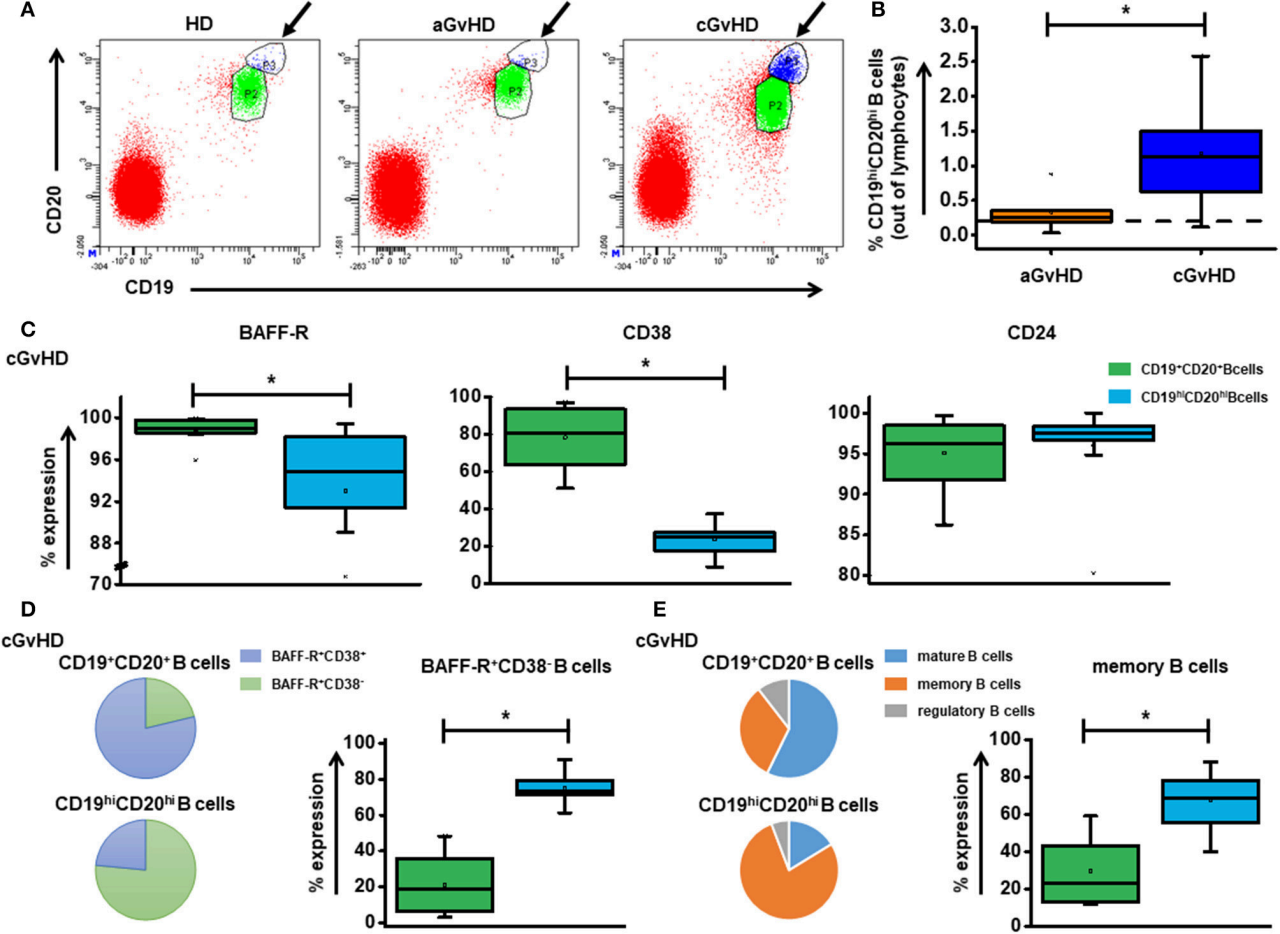
The role of CD19^hi^CD20^hi^ B cells in cGvHD. **(A)** shows representative dot plots of CD19^hi^CD20^hi^ B cells among HD, aGvHD and cGvHD groups. **(B)** displays the frequency of CD19^hi^CD20^hi^ B cells in both aGvHD and cGvHD groups prior to ECP treatment. **(C)** Characterization of CD19^hi^CD20^hi^ B cells showed significantly lower expression of BAFF-R and CD38 but slightly increased CD24 expression. **(D,E)** When compared to CD19^+^CD20^+^ B cells, CD19^hi^CD20^hi^ B cells showed a different component pattern, a significantly higher BAFF-R^+^CD38^−^ proportion and memory B cells. Dashed lines represent the corresponding median value of frequencies observed in 25 HDs. Differences in cell frequency between different groups were assessed by Independent *T* test. In all tests, a *p*-value < 0.05 was considered to be statistically significant. ^*^means *p* < 0.05.

Strikingly, a novel CD19^hi^CD20^hi^ B cell population is significantly elevated in cGvHD patients (Figures 2A,B), suggesting a crucial role in the development of cGvHD. A reduced expression was observed for BAFF-R (*p* = 0.007) and CD38 (*p* = 0.000) on CD19^hi^CD20^hi^ B cells (Figure 2C) with consequently a significantly higher percentage of BAFF-R^+^CD38^−^ B cell subset (Figure 2D) and CD24^+^CD38^−^ memory B cell subset (Figure 2E) when compared with CD19^+^CD20^+^ B cells. Under ECP therapy, patients with either stable cGvHD or with response tended to have a decrease in frequency of CD19^hi^CD20^hi^ B cells, whereas an increase was observed in patients with progressive cGvHD (Supplementary Figure 1).

### Education of MDSCs by ECP therapy

**Figure 3:**
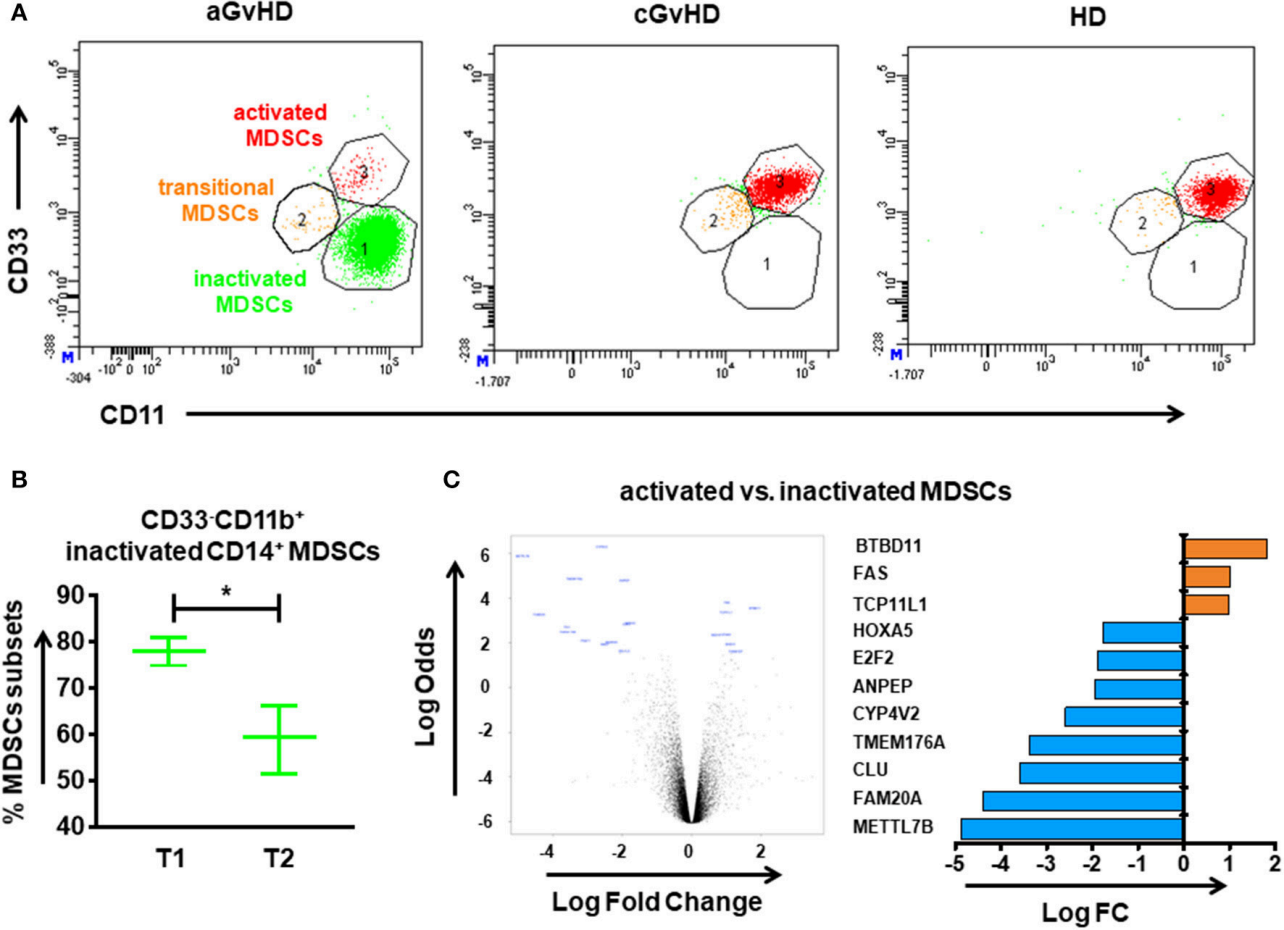
CD14^+^HLA-DR^−/low^ MDSC subpopulations in the peripheral blood of GvHD patients with ECP treatment. The immunophenotype of MDSCs was assessed by flow cytometry. **(A)** Different components of inactivated, transitional and activated subsets were observed within CD14^+^HLA-DR^−/low^ MDSCs among aGvHD patients, cGvHD patients and healthy donors (HDs) suggesting a development from inactivated into activated MDSCs. **(B)** A reduction of inactivated MDSCs was observed after ECP therapy in aGvHD patients. **(C)** The volcano plot shows the gene expression between activated MDSCs and inactivated MDSCs. The horizontal axis represents the fold change in intensity and the vertical axis represents statistical significance (Log Odds). The bar chart indicates the differential gene expression between activated and inactivated MDSCs. Gene expression was assessed after adjustment by the Benjamini-Hochberg procedure. Differences in cell frequency between different groups were assessed by paired-sample *T* test. In all tests, a *p*-value < 0.05 was considered to be statistically significant. ^*^means *p* < 0.05.

Inactivated CD33^−^CD11b^+^, transitional CD33^dim^CD11b^dim^, and activated CD33^+^CD11b^+^ subsets were identified out of CD14^+^HLA-DR^−/low^ MDSCs. Figure 3A depicts the different components of inactivated, transitional and activated subsets within CD14^+^HLA-DR^−/low^ MDSCs among aGvHD, cGvHD patients and HDs, suggesting a development from inactivated to activated MDSCs. After 8 cycles of ECP therapy, the inactivated subset which is absent in HDs could be dramatically decreased in aGvHD patients (Figure 3B). In cGvHD cohort, the homogeneous CD14^+^HLA-DR^−/low^ MDSC population, activated CD33^+^CD11b^+^ subset, with a normalization was observed (data not shown).

In order to determine the impact of ECP therapy on gene expression profiling of MDSCs, we performed microarray analysis in the highly purified two different subpopulations of MDSCs from HD as well as aGvHD patients. Relative to inactivated MDSCs, three genes were significantly upregulated, whereas eight genes were significantly downregulated in activated MDSCs (Figure 3C). Among these 11 genes, FAS, which positively regulates lymphocyte and inflammatory cell apoptotic process, as well as MAPK cascade and myeloid cell differentiation, while has a negative regulation on B cell activation, showed significantly higher expression in activated MDSCs. In addition, pathways related to negative regulation of immune system process were enriched in activated MDSCs (Supplementary Table 2). Taken together, our data indicated activated MDSCs have stronger immunosuppressive potency.

### Kinetics of different regulatory cells

**Figure 4:**
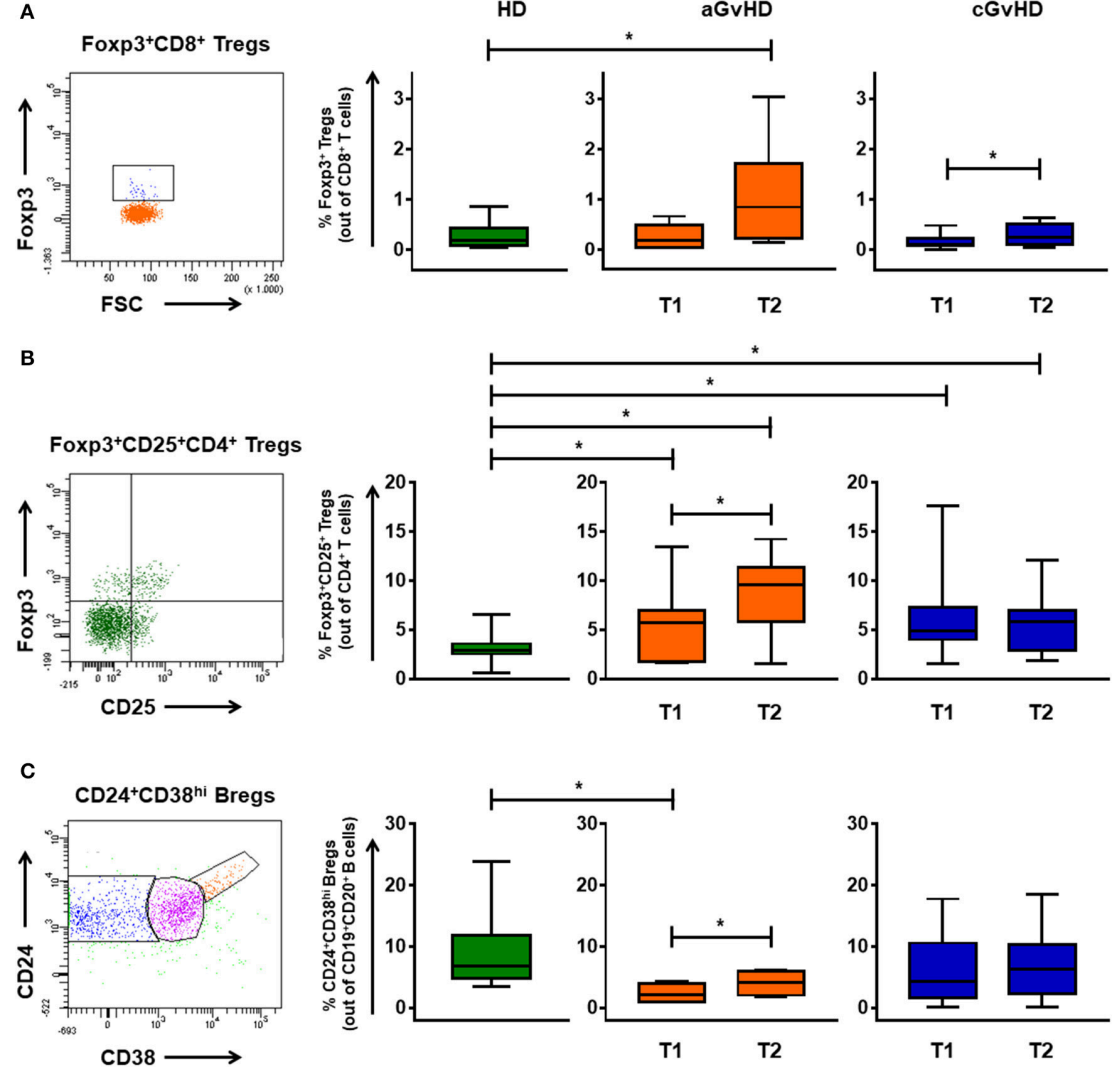
Immunomodulation of regulatory T and B cells through ECP. The percentages of CD8^+^ Tregs **(A)**, FoxP3^+^CD25^+^CD4^+^ Tregs **(B)**, and CD24^+^CD38^hi^ Bregs **(C)** were monitored in patients with aGvHD and cGvHD before and after ECP therapy. Foxp3^+^CD8^+^ Tregs significantly increased under ECP therapy in both aGvHD and cGvHD patients, along with significant up-regulation of Foxp3^+^CD4^+^ Tregs and Bregs in aGvHD patients, as assessed by paired-sample *T* test. ^*^ means *p* < 0.05.

Besides MDSCs, also FoxP3^+^CD8^+^ and FoxP3^+^CD25^+^CD4^+^ Tregs, and CD24^+^CD38^hi^ regulatory B cells (Bregs) were identified to be of relevance in our cohort (Figure 4). In the aGvHD cohort, the frequency of CD8^+^ Tregs in patients before ECP treatment was similar to the values of HDs (Figure 4A). However, it was significantly increased by ECP therapy when compared to HDs. Consistent with previous reports (9, 10, 11, 13), a significant increase of CD4^+^ Tregs was seen after ECP therapy (Figure 4B). Moreover, ECP could increase Bregs to reach the HD values (Figure 4C). In contrast, the frequency of CD4^+^ Tregs and Bregs was barely influenced by ECP therapy in cGvHD patients (Figures 4B,C). However, the frequency of CD8^+^ Tregs in cGvHD was apparently elevated under ECP treatment (Figure 4A).

### Reduction of proinflammatory cytokines

**Figure 5:**
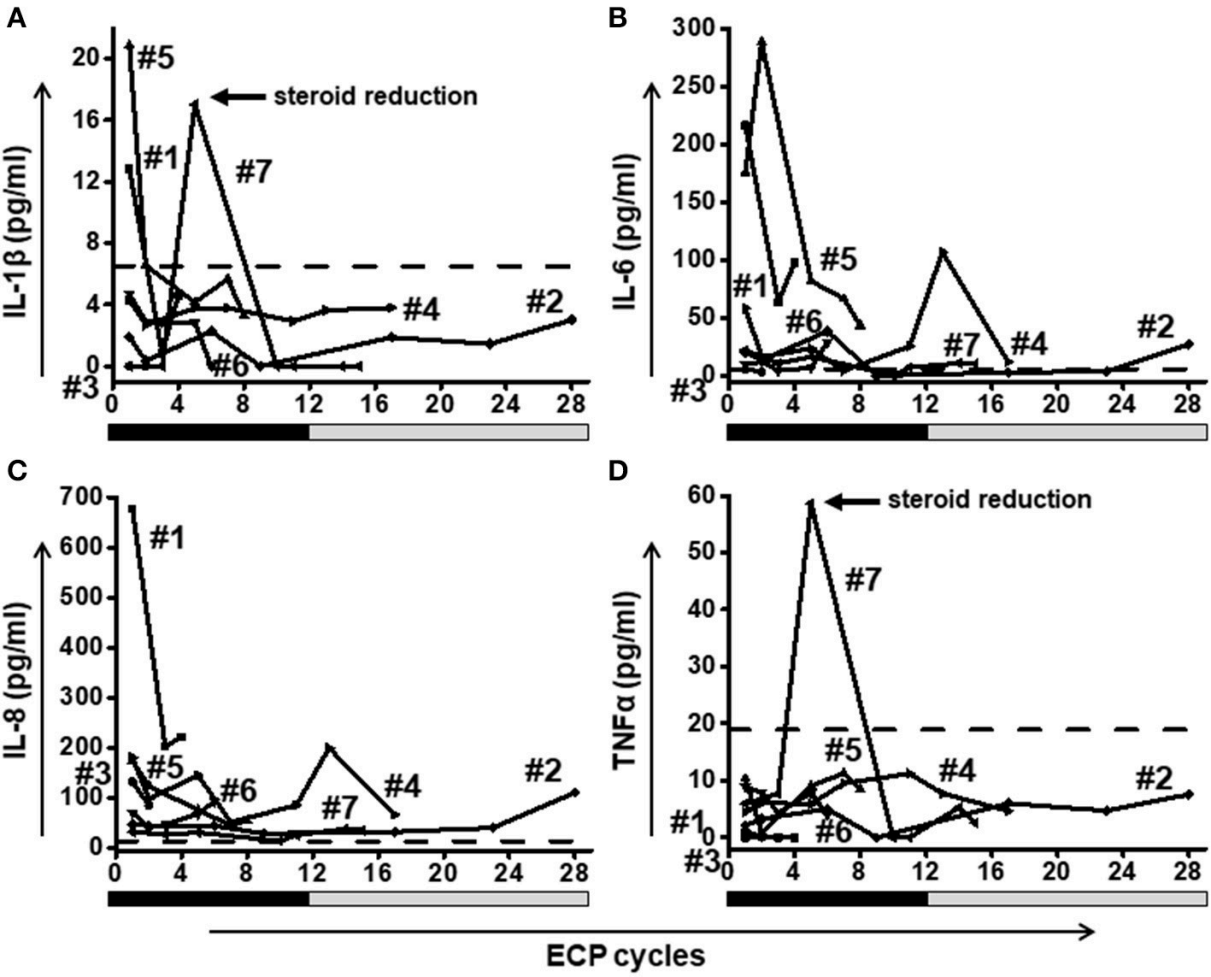
A fast reduction of proinflammatory cytokines IL-1β **(A)**, IL-6 **(B)**, IL-8 **(C)**, and TNF-α **(D)** was observed in all patients. Patient #7 showed a rebound of IL-1β and TNF-α after rapid steroid reduction. Eventually the level of both cytokines decreased when ECP was continued. Dashed lines represent the corresponding median value of cytokine levels observed in healthy donors. The frequency of ECP cycles is indicated on the x-axis. The black bars below the x-axis indicate a high frequency of ECP treatment during the first 12 weeks (twice per week) followed by a gray bar representing a reduced frequency (twice every second week) in weeks 13–28.

Elevated levels of proinflammatory cytokines (IL-1β, IL-6, and IL-8) were found even on high doses of steroids e.g., 2 mg/kg body weight. ECP had a positive effect in all cases with a decline of proinflammatory cytokines (Figure 5). High peaks of cytokines IL-1β (Figure 5A) and TNF-α (Figure 5D) in patient #7 caused by rapid steroid reduction were returned back to low levels by ongoing ECP treatment.

### Intact anti-viral and anti-leukemia immunity under ECP

**Figure 6:**
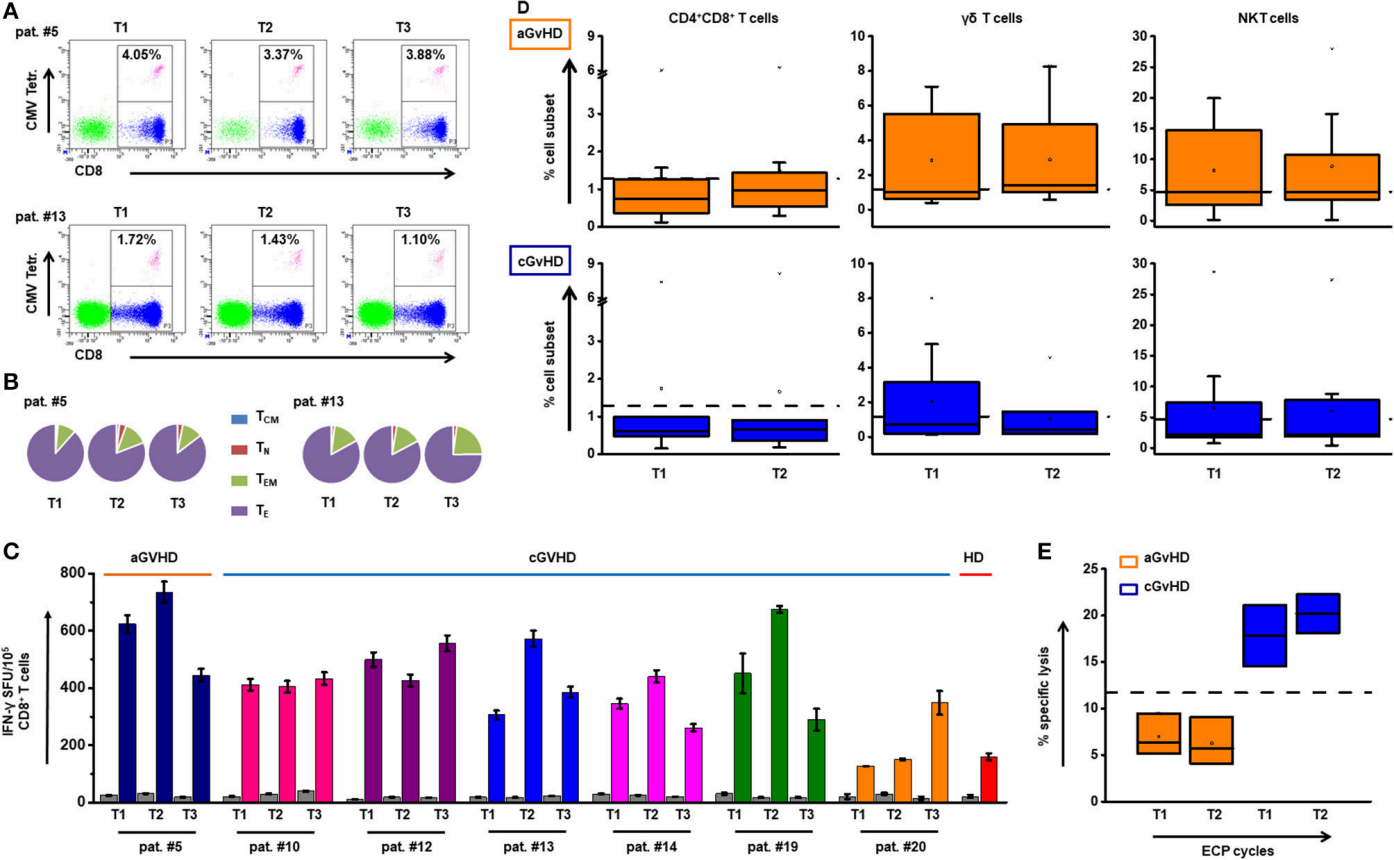
Impact of ECP therapy on anti-viral and anti-leukemic immune responses. **(A)** The representative dot plots with the frequency of CMV-specific CD8^+^ T cells are shown at different time (T) points before (T1) and after (T2 and T3) ECP treatment in aGvHD patient #5 and cGvHD patient #13. **(B)** The distribution of T_CM_, T_N_, T_EM_, and T_E_ within the CMV specific CD8^+^ T cells in patients #5 and #13 is indicated. **(C)** The secretion of IFN-γ by virus specific T cells was measured by IFN-γ ELISpot assay. The bar chart shows the overview of the INF-γ secretion by CD8^+^ T cells in seven patients under ECP treatment. There is no significant difference among T1, T2, and T3 (*p* ≥ 0.05, one-way ANOVA test). Under ECP treatment, the frequency of CMV-specific CD8^+^ T cells was maintained. Most cells were T_E_ cells followed by T_EM_ cells. The function of these cells in terms of IFN-γ release kept stable. **(D)** The dynamic changes of CD4^+^CD8^+^ T cells, γδ T cells and NKT cells in aGvHD (upper panel) and cGvHD patients (lower panel) under the ECP treatment. Cell frequencies were not significantly different between before and under ECP treatment groups, which assessed by Paired sample *T* test. **(E)** A 4-h ^51^Cr release assay was performed to test the NK activity, which was calculated by the following formula: % specific lysis = [c.p.m. (experimental release)–mean c.p.m. (spontaneous release)]/[mean c.p.m. (maximal release)–mean c.p.m. (spontaneous release)] × 100. The box chart shows the NK activity against K562 cells at two different time points in aGvHD group and cGvHD group. There was no significant difference, as assessed by Paired sample *T* test. Each box represents three independent patients. The NK cell function was stable over the time of ECP treatment. The dashed lines represent the corresponding median value of frequencies observed in 25 healthy donors. In all tests, a *p* < 0.05 was considered to be statistically significant.

Neither frequency nor IFN-γ release of virus specific T cells was hampered by ECP (Figures 6A–C). Besides these, no significant influence of ECP therapy on CD4^+^CD8^+^ T cells, γδ T cells, and NKT cells as other well-established protective cell subsets could be observed (Figure 6D). Furthermore, no significant change in NK activity was shown during ECP (Figure 6E).

### Dissection of cell population dynamics with medicines and clinical parameters

**Figure 7:**
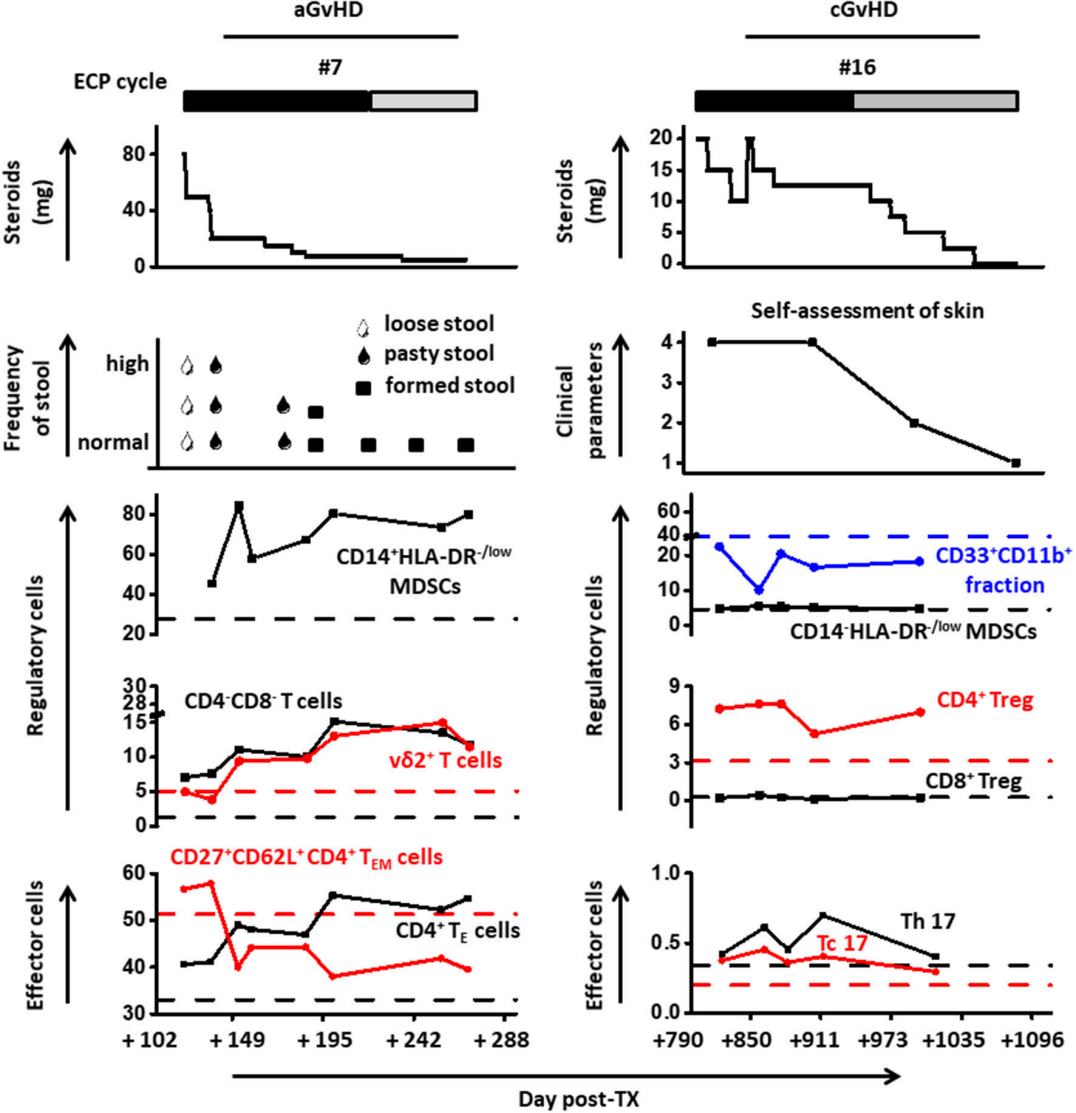
Cell population dynamics are displayed with steroid dosing and clinical parameters in representative patients under ECP therapy.

To further corroborate our findings and to investigate the underlying mechanism of ECP therapy in GvHD, a comprehensive analysis including immunosuppressive medicines as well as cell populations was performed in representative patients (Figure 7). Immunosuppressive therapy was reduced or stabilized following ECP therapy with clinical improvement, including a decrease of stool frequency and change of consistency from loose to formed stools (patient #7), as well as alleviation of itching, dryness and pain of the skin (patient #16), as shown in Figure 7.

Activated MDSCs subset was steadily increased under ECP therapy in patient #7. The frequency of DN T cells was apparently elevated under ECP treatment (patient #7: 1.2-fold). Similar changes were detected for vδ2^+^ T cells (patient #7: 2-fold). Furthermore, a strong up-regulation of CD4^+^ T_E_ cells with a dramatical loss of homing marker CD62L was observed under therapy. The frequency of MDSCs and Tregs in patient #16 was stable over time. However, a rebound of Th17 after steroid reduction could be decreased when ECP was continued.

## Discussion

Allo-HSCT constitutes a curative therapy for many hematological malignancies. Its success depends on the engraftment of donor hematopoietic stem cells and on the complete reconstitution of a donor-derived immune system in the recipient (23). However, unbalanced reconstitution of diverse stimulatory and suppressive elements may lead to expansion of alloreactive T cells and contribute to the development of GvHD. Simultaneously, GvL is mediated by donor effector T cells and results in a reduced risk of relapse through the elimination of MRD (24). Thus, after allo-HSCT the major goal is to control the activation of alloreactive T cells and to mitigate, but not to completely prevent, GvHD through the administration of immunosuppressive drugs.

Steroids with strong immunosuppressive and anti-inflammatory effects remain the gold standard for initial management of GvHD (15). However, steroids hamper not only effector T cells but also regulatory cells, thus indicating an inhibition of tolerance induction through immunosuppressors (25). Moreover, high doses of steroids are usually accompanied by an increased risk for infections and subsequent mortality (14). Therefore, successful management of GvHD does not in general imply high-dose immunosuppression but rather a balance between effector and regulatory cells. In the case of steroid-refractory GvHD the treatment is rather heterogeneous. As an optional second line treatment, ECP shows promising clinical responses and a steroid-sparing effect, which seems to be favorable in the treatment of GvHD as several studies reported (6, 26–28).

B cells contribute to the pathogenesis of both aGvHD and cGvHD due to their effect on host APCs (24, 29). When analyzing B cells in our cohort, we observed a clear CD19^hi^CD20^hi^ B cell population in cGvHD patients and demonstrated that BAFF-R^+^CD38^−^ memory B cells represent the predominant subset within CD19^hi^CD20^hi^ B cells. These memory B cells could be rapidly reactivated, leading to the production of large quantities of high-affinity antigen-specific antibodies (30). Moreover, CD38^−^ memory B cells have the potency to produce pro-inflammatory cytokine TNF-α (31). BAFF, which is significantly increased in plasma of cGvHD patients, could further activate B cells via binding to BAFF receptor (32, 33). Taken together, we assumed CD19^hi^CD20^hi^ B cells might play a role in the cGvHD pathophysiology. Subsequently, the hypothesis was confirmed by our finding that a reduction of CD19^hi^CD20^hi^ B cells was observed in patients with clinical improvement under ECP treatment.

Modulation of the trafficking patterns of alloreactive T cells and NK cells has been identified as an effective way of ameliorating GvHD. The important role of L-selectin in effector cell migration to GvHD target tissues has been recently demonstrated (24, 34). Our data indicate that ECP promotes the NK cell differentiation from CD56^bri^ to CD56^dim^ NK cells with loss of expression of NKG2D and CD62L, but without hampering the GvL effect. This is substantiated by our own data and a previous report showing that CD62L^−^CD56^dim^ NK cells exert high cytotoxicity against MHC class I negative tumor target cells (35). Moreover, we found a loss of CD62L in T_E_ population as well. CD62L^−^ T cells not only facilitate hematopoietic engraftment and contribute to phenotypic and functional T cell reconstitution after transplantation without causing GvHD, but also enhance functional immune reconstitution against tumor and viral antigens, which was recently reported by Zhang et al. (36).

Regulatory cells are known to promote immune tolerance after allo-HSCT and to shift the balance toward graft survival and away from GvHD. The importance of FoxP3^+^CD4^+^ Tregs in ECP-induced tolerance is well established. In accordance with other previous results (9–11, 13), our data show a significant increase of FoxP3^+^CD25^hi^CD4^+^ Tregs under ECP therapy in aGvHD patients. Apart from FoxP3^+^CD4^+^ Tregs, highly immunosuppressive FoxP3^+^CD8^+^ Tregs which display preferential tropism for the gastrointestinal tract and inhibit class I-restricted immune responses (37), were dramatically increased in both aGvHD and cGvHD patients after ECP therapy. Bregs have a prominent immunoregulatory potential as well as broad inhibitory effect on CD4^+^ T cells and monocytes. Thus, it can effectively prevent GvHD through inhibiting Th1 and Th17 differentiation and expanding CD4^+^ Tregs without adverse effect on GvL activity (38–47). Our data show a significant increase of Bregs in aGvHD patients.

Immunosuppressive MDSCs, especially the neutrophilic subgroup, can be augmented by ECP and thereby hamper Th1 and Th17 cells through metabolism of L-arginine (12). In our study, an increase in monocytic MDSCs was observed in aGvHD patients. Notably, our data further demonstrate that ECP may promote the development of MDSCs by switching the inactivated (CD33^−^CD11b^+^CD14^+^HLA-DR^−/low^) to activated (CD33^+^CD11b^+^CD14^+^HLA-DR^−/low^) subsets. In activated MDSCs we observed an up-regulation of the suppressive FAS gene together with an enrichment of the pathway related to negative regulation of immune system process. It suggests these activated cells have a more potent immunosuppressive capacity thus contributing to immune tolerance.

Proinflammatory cytokines (IL-1β, IL-6, IL-8, and TNF-α) play an important role in all phases of GvHD pathophysiology from the activation of host antigen presenting cells, priming of immune effector cells, to tissue damage mediated by cytotoxic effector cells (48–50). Conditioning therapy induced-intestinal damage is the crucial step in the initiation phase of acute gastrointestinal GvHD where the inflamed gut mucosa aggravates the activation of alloreactive T cells (51). IL-1β plays a crucial role herein: (i) low level of IL-1β could protect the gut mucosa from damage during the early stages of inflammation (52), while (ii) increased level of IL-1β is associated with ongoing and progressive mucosal inflammation in acute gastrointestinal GvHD, which contributes to a state of uncontrolled inflammation and progressive tissue damage (53–56). Moreover, the injured tissue could release other proinflammatory cytokines (such as IL-6 and TNF-α), which activate and amplify the response of alloreative donor T cells (57). IL-6 not only enhances the development of Th17 and Tc17 cells but also inhibits the development of Tregs (58, 59). And TNF-α has the ability to enhance CD8^+^ T cell mediated alloreactivity exacerbating immune destruction of GvHD target tissues (60). Recent study showed that blocking TNF-α signaling may protect donor hematopoietic stem cells and progenitors from the host's detrimental inflammatory signals after transplantation (61). Therefore, TNF-α inhibitory drugs such as the monoclonal antibodies infliximab and adalimumab are now in use in the clinic for the treatment patients with steroid-refractory GvHD. IL-8 contributes to downstream tissue damage and transplant-related mortality and has been defined as a diagnostic and prognostic biomarker for aGvHD (62, 63). In the light of our data, steroids were insufficient to reduce proinflammatory cytokines in all cases since some patients still had high levels of cytokines under the high-dose of immunosuppressive medication. By contrast, ECP has a potent effect on down-modulation of proinflammatory cytokines.

In line with previous studies (64, 65), our patients showed neither increased susceptibility to infections, nor reactivation of CMV under ECP therapy. On the cellular level, the frequency of cytotoxic CD8^+^ T cells, the most important mediators of GvL activity, as well as CD4^+^CD8^+^ T cells, γδ T cells, and NKT cells remained constant under ECP therapy. In addition, CMV specific CD8^+^ T cells were maintained under ECP. Particularly, ECP did neither affect NK activity nor the capacity of virus-specific CD8^+^ T cells to produce IFN-γ. Our results suggest that ECP therapy preserves immunity against infections as well as the GvL effect via maintaining the quality and quantity of effector cells.

**Figure 8:**
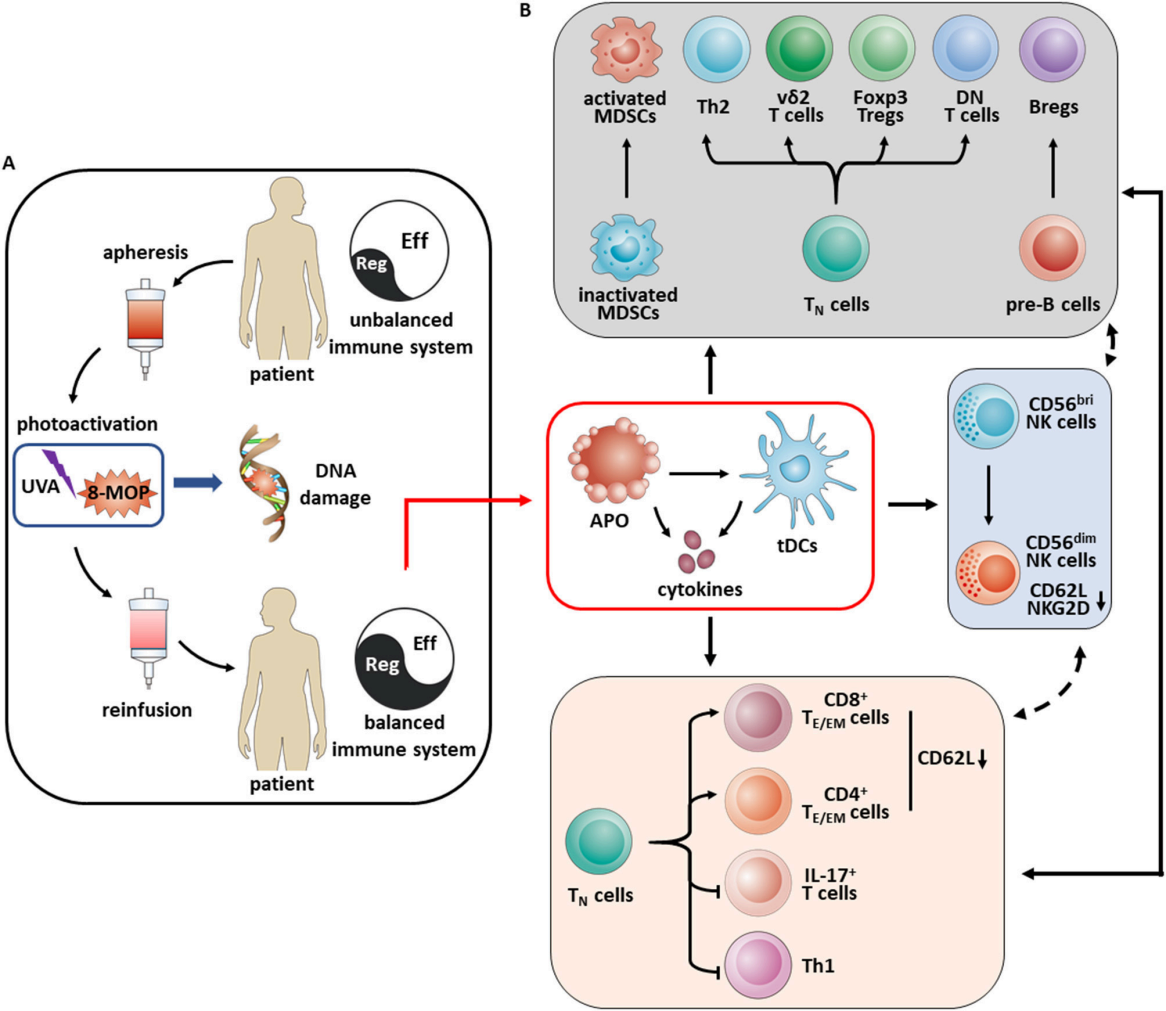
Schematic overview of mechanisms of immunomodulation in aGvHD patients under ECP therapy. **(A)** ECP is a cell-based immunotherapy, involving (i) apheresis with *ex vivo* collection of peripheral mononuclear cells, (ii) photoactivation with exposure of leukocyte-enriched plasma to the photosensitizing agent 8-methoxypsoralen and ultraviolet A light which results in crosslinking of the pyrimidine bases in DNA leading to cell death through apoptosis, (iii) reinfusion of the ECP-treated cells to the patient. **(B)** Apoptosis of ECP-treated cells play a key role *in vivo*. Engulfing these apoptotic cells by immature dendritic cells results in a tolerogenic phenotype and promotes tolerance through the secretion of immunosuppressive cytokines such as IL-10 and TGF-β as well. Upregulation of activated MDSCs, Th2, vδ2^+^ T cells, FoxP3^+^ Tregs, double negative (DN) T cells and Bregs result in an overall increase in immune tolerance, accompanied by a decrease of immune effector cells like IL-17^+^ T cells and Th1 cells as well as education of T_E/EM_ cells via decreasing CD62L expression. Besides these, ECP promotes the NK cell differentiation from CD56^bri^ to CD56^dim^ NK cells with loss of expression of NKG2D and CD62L.

Taken together, the key findings of our study indicate that (1) under ECP treatment steroids could be significantly reduced. This will make patients less prone to opportunistic diseases. (2) Newly defined CD19^hi^CD20^hi^ B cells, potentially contributing to cGvHD, could be significantly reduced under ECP treatment. (3) Expression of CD62L on NK cells was significantly down-regulated by ECP treatment. In addition, ECP could also modulate CD62L expression on CD4 T_E_ cells. This indicates less migration of effector cells into the site of inflammation. (4) The frequency of Foxp3^+^CD8^+^ Tregs, Foxp3^+^CD4^+^ Tregs, and Bregs was significantly elevated. The elevated frequency of regulatory cells will also hamper inflammatory processes. (5) Levels of proinflammatory cytokines were significantly reduced in patients under ECP therapy. (6) ECP treatment had no significant impact on anti-viral/anti-leukemic effect. This will make patients less prone to reactivation of viral disease and relapse of the underlying hematological neoplasm. A summary of the key findings of this work as well as of previous studies (27, 28, 66–68) is depicted in Figure 8.

Therefore, ECP constitutes an effective immunomodulatory therapy for both aGvHD and cGvHD without hampering anti-viral and anti-leukemic effects.

## Author contributions

AS, MS, and LW designed the research. LW, MN, J-MH, CK, and VE performed the experiments. AS, MS, TL, UH, SS, PW, WK, and PD treated the patients. LW, BN, and AS acquired and analyzed the data. LS and MaS performed the biostatistics. MS, AS, LW, AH-K, BC, AH, PD, CM-T, IH, RY, AN, and WK discussed the organization of the manuscript. LW wrote the manuscript. All authors critically reviewed the manuscript. MS, AS, PD, CM-T, IH, and AN edited the manuscript. MS and AS supervised the work.

### Conflict of interest statement

PW Honoraria and membership on Advisory Boards for Sanofi-Aventis. This Investigator Initiated Research was funded by Therakos, Inc., a Mallinckrodt Pharmaceuticals company. The remaining authors declare that the research was conducted in the absence of any commercial or financial relationships that could be construed as a potential conflict of interest.
