# Supplementary material for: Modulation of B Cells and Homing Marker on NK Cells Through Extracorporeal Photopheresis in Patients With Steroid-Refractory/Resistant Graft-Vs.-Host Disease Without Hampering Anti-viral/Anti-leukemic Effects
Source: Front Immunol. 2018 Oct 8;9:2207. doi: 10.3389/fimmu.2018.02207 (PMC6186805; doi:10.3389/fimmu.2018.02207)
Supplement: Supplementary file 1 [file Table_1.docx]

Supplementary Material

Modulation of B cells and homing marker on NK cells through extracorporeal photopheresis in patients with steroid-refractory/resistant graft-versus-host disease without hampering anti-viral/anti-leukemic effects

Lei Wang^1^, Ming Ni^1,2^, Angela Hückelhoven-Krauss ^1^, Leopold Sellner^1^, Jean-Marc Hoffmann^1^, Brigitte Neuber^1^, Thomas Luft^1^, Ute Hegenbart^1^, Stefan Schönland^1^, Christian Kleist^3^, Martin Sill^4^, Bao-an Chen^5^, Patrick Wuchter^1,6^, Volker Eckstein^1^, William Krüger^7^, Inken Hilgendorf^8^, Ronit Yerushalmi^9^, Arnon Nagler^9^, Carsten Müller-Tidow^1^, Anthony D. Ho^1^, Peter Dreger^1^, Michael Schmitt^1^, Anita Schmitt^1*^

*** Correspondence:** PD Dr. med. Anita Schmitt: anita.schmitt@med.uni-heidelberg.de

# Supplementary Table 1. Antibody list.

| **Name** | **Dye** | **Isotype** | **Clone** | **V_Working_ (μl)** | **Company** | **Cat.NO.** |
| --- | --- | --- | --- | --- | --- | --- |
| CD3 | V500 | Mouse IgG1(κ) | UCHT1 | 4 | BD | 561416 |
| CD3 | PerCP | Mouse IgG1(κ) | SK7 | 1 | Biolegend | 344814 |
| CD3 | Pacific Blue | Mouse IgG2a(κ) | HIT3a | 2 | Biolegend | 300330 |
| CD4 | FITC | Mouse IgG1(κ) | RPA-T4 | 1 | Biolegend | 300506 |
| CD8 | PerCP | Mouse IgG1(κ) | SK1 | 0.5 | Biolegend | 344708 |
| CD11b | APC | Mouse IgG1(κ) | ICRF44 | 2 | Biolegend | 301310 |
| CD14 | APC-eFluor780 | Mouse IgG1(κ) | 61D3 | 2 | eBioscience | 47-0149-42 |
| CD16 | APC-Cy7 | Mouse IgG1(κ) | 3G8 | 0.5 | Biolegend | 302018 |
| CD19 | PE | Mouse IgG1(κ) | HIB19 | 0.5 | Biolegend | 302208 |
| CD20 | PerCP-Cy5.5 | Mouse IgG1(κ) | L27 | 10 | BD | 332781 |
| CD24 | PE-Cy7 | Mouse IgG1(κ) | eBioSN3 | 2 | eBioscience | 25-0247 |
| CD25 | PE-Cy7 | Mouse IgG1(κ) | BC96 | 4 | Biolegend | 302612 |
| CD27 | FITC | Mouse IgG1(κ) | M-T271 | 2.5 | BD | 555440 |
| CD33 | FITC | Mouse IgG1(κ) | HIM3-4 | 5 | BD | 555626 |
| CD38 | APC | Mouse IgG1(κ) | HIT2 | 15 | BD | 555462 |
| CD45RA | APC | Mouse IgG2b(κ) | HI100 | 2 | Biolegned | 304112 |
| CD56 | Alexa 488 | Mouse IgG1(κ) | HCD56 | 2 | Biolegend | 318312 |
| CD62L | eFluor@450 | Mouse IgG1(κ) | DREG-56 | 1 | eBiocience | 48-0629 |
| CD197 | PE-Cy7 | Rat IgG2a(κ) | 3D12 | 4 | eBioscience | 25-1979 |
| CD314 | PE | Mouse IgG1(κ) | 1D11 | 1 | Biolegend | 320806 |
| HLA-DR | PerCP | Mouse IgG2a(κ) | L243 | 2 | eBioscience | 9043-9952-120 |
| vσ2 TCR | PE | Mouse IgG1(κ) | B6 | 1 | Biolegend | 331408 |
| γσ TCR | APC | Mouse IgG1(κ) | B1 | 4 | Biolegend | 331212 |
| FoxP3 | PE | Mouse IgG1(κ) | 259D | 4 | Biolegend | 320208 |
| IL17a | Alexa647 | Mouse IgG1(κ) | BL168 | 2 | Biolegend | 512310 |

Abbreviations: Vworking = working volume of the respective antibody; Cat. No. = catalogue number; PerCP = peridinin chlorophyll; FITC = fluorescein isothiocyanate; APC = allophycocyanin; PE = phycoerythrin; PE-Cy7 = phycoerythrin-Cyanin 7.

# Supplementary Table 2. Pathway enrichment of activated MDSCs.

| NO. | GO.ID | Term Annotated |
| --- | --- | --- |
| # 1 | GO:0032462 | regulation of protein homooligomerization |
| # 2 | GO:1903706 | regulation of hematopoiesis |
| # 3 | GO:1903707 | negative regulation of hematopoiesis |
| # 4 | GO:0002683 | negative regulation of immune system process |
| # 5 | GO:0032459 | regulation of protein oligomerization |
| # 6 | GO:0097028 | dendritic cell differentiation |
| # 7 | GO:1902105 | regulation of leukocyte differentiation |
| # 8 | GO:1902106 | negative regulation of leukocyte differentiation |
| # 9 | GO:1904019 | epithelial cell apoptotic process |
| # 10 | GO:0006631 | fatty acid metabolic process |

# Supplementary Figure legend

**Supplementary Figure 1**. The role of CD19^hi^CD20^hi^ B cells in cGvHD. It depicts dynamic changes of CD19^hi^CD20^hi^ B cells under ECP treatment in the group of cGvHD patients. Dashed lines represent the corresponding median value of frequencies observed in 25 HDs. The frequency of ECP cycles is indicated on the x-axis. The black bars below the x-axis indicate a high frequency of ECP treatment followed by a gray bar representing a reduced frequency. PR, partial response, was defined as improvement in a measure for at least one organ site without progression in measures for any other organ site. Patients with stable disease (SD) showed no or only a slight improvement of their clinical symptoms, whereas the steroids could be reduced. PR*: the cGvHD patients showed an initial response to the ECP treatment but during further treatment the clinical situation aggravated again. Differences in cell frequency between different groups were assessed by independent T test. In all tests, a *p*-value < 0.05 was considered to be statistically significant. * means p < 0.05.
