# Supplementary figures and images for: Modulation of B Cells and Homing Marker on NK Cells Through Extracorporeal Photopheresis in Patients With Steroid-Refractory/Resistant Graft-Vs.-Host Disease Without Hampering Anti-viral/Anti-leukemic Effects
Source: Front Immunol. 2018 Oct 8;9:2207. doi: 10.3389/fimmu.2018.02207 (PMC6186805; doi:10.3389/fimmu.2018.02207)

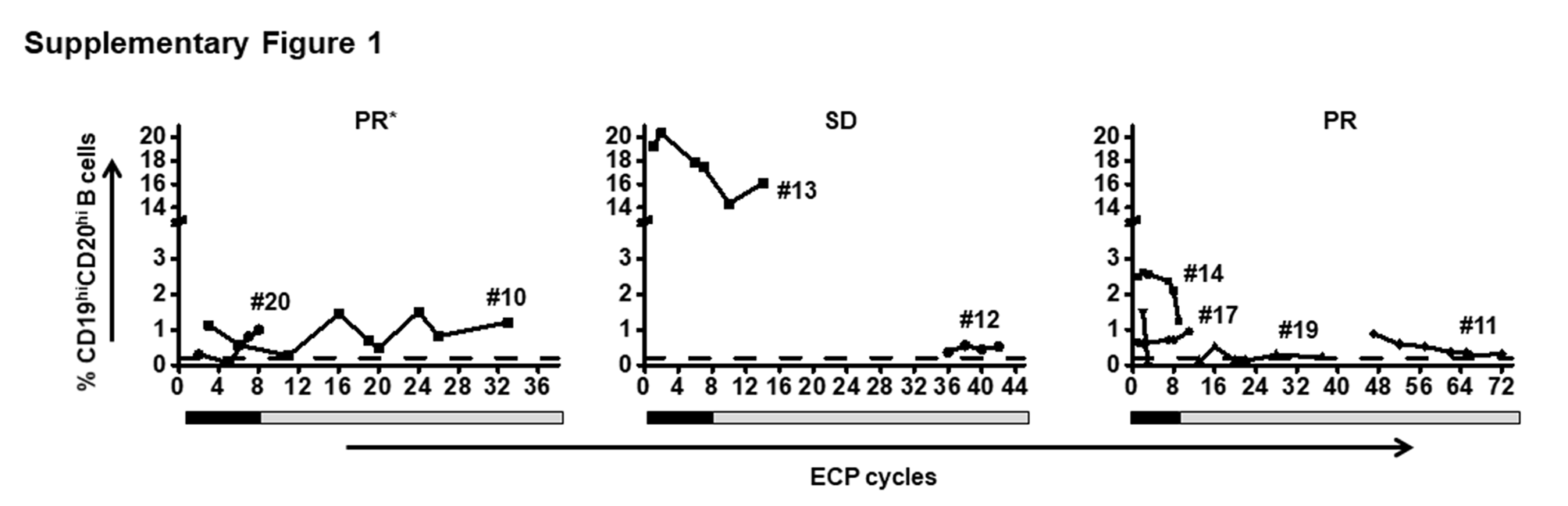

Supplement: Supplementary file 2 [file Image_1.TIF]
